# The Association Between Erythropoiesis Resistance Index and Clinical Outcomes in Hemodialysis Patients: A Nationwide Study

**DOI:** 10.3390/jcm14082812

**Published:** 2025-04-18

**Authors:** Seok-Hui Kang, So-Young Park, Yu-Jeong Lim, Bo-Yeon Kim, Ji-Young Choi, Jun-Young Do, A-Young Kim

**Affiliations:** 1 Division of Nephrology, Department of Internal Medicine, College of Medicine, Yeungnam University, Daegu 42415, Republic of Korea; kangkang@ynu.ac.kr (S.-H.K.);; 2 Department of Physiology, College of Medicine, Yeungnam University, Daegu 42415, Republic of Korea; 3 Quality Assessment Department, Health Insurance Review and Assessment Service, Wonju 26465, Republic of Korea

**Keywords:** erythropoiesis-stimulating agent, hemodialysis, mortality, erythropoiesis resistance

## Abstract

**Background:** Although erythropoiesis-stimulating agent (ESA) therapy is fundamental for correcting anemia, excessive ESA administration is associated with increased risks. This study aimed to investigate the impact of the erythropoietin resistance index (ERI) on clinical outcomes in a population-based cohort of hemodialysis (HD) patients. **Methods:** This retrospective study analyzed datasets from patients who underwent periodic HD quality assessments and their claims data. Overall, we included 35,913 patients. Participants were divided into quartiles based on the ERI during the 6-month assessment period: Q1, Q2, Q3, and Q4 groups. **Results:** The 5-year survival rates were 68.8% (Q1), 67.8% (Q2), 66.9% (Q3), and 60.2% (Q4) (*p* < 0.001). Multivariable analysis showed the same trends as the univariable analysis. Additionally, a spline curve using the multivariable model indicated that the increased ERI was linked to all-cause mortality. However, cardiovascular events were not associated with ERI quartiles in Cox regression analyses. Subgroup analysis revealed that in most subgroups, the all-cause mortality was significantly higher in those with a high ERI than in those with a low ERI. Further analysis using the balanced cohort, which attenuated baseline characteristic differences, confirmed that the high mortality in those with a high ERI was maintained. **Conclusions:** Our population-based cohort study reveals an association between the ERI and all-cause mortality in HD patients.

## 1. Introduction

Chronic kidney disease or acute kidney injury can progress to end-stage kidney disease (ESKD), which is treated with dialysis or kidney transplantation. Hemodialysis (HD) is the most widely used dialysis modality among the three renal replacement therapies worldwide [1]. The survival and overall prognosis of HD patients are significantly poorer compared to the general population [2]. Therefore, identifying various risk factors associated with poor outcomes in these patients is crucial. Patients with ESKD frequently develop anemia due to functional and/or absolute iron deficiency, as well as erythropoietin deficiency [3]. Such anemia increases cardiac workload and elevates blood pressure and sympathetic activity, contributing to the development and progression of various cardiovascular diseases (CVDs) [4]. Thus, proper correction of anemia is essential for reducing mortality and CVD risk in HD patients. Recent clinical guidelines have emphasized the importance of maintaining target hemoglobin levels at 10–11.5 g/dL [5]. However, beyond simply achieving the target hemoglobin concentration, identifying novel prognostic indicators associated with anemia correction could provide valuable insights for optimizing anemia management and implementing effective interventions to improve patient outcomes.

Erythropoiesis-stimulating agents (ESAs) are the cornerstone of anemia treatment in HD patients. Although ESA therapy is fundamental for correcting anemia, excessive ESA administration is associated with increased risk [6]. ESA therapy can be quantified using the erythropoietin resistance index (ERI), an important marker reflecting ESA responsiveness. Recent studies have reported an association between the ERI and mortality, as well as other complications, in HD patients [7,8,9]. However, large-scale studies are needed to further establish ERI as a reliable prognostic indicator. Consequently, this study aimed to investigate the impact of ERI on mortality and cardiovascular events (CVEs) in a population-based cohort of HD patients. The hypothesis is that higher ERI quartiles could independently predict increased all-cause mortality but not CVEs.

## 2. Materials and Methods

### 2.1. Data Sources and Participants

This retrospective study analyzed datasets from patients who underwent periodic HD quality assessments and their claims data [10,11]. Briefly, the first HD quality assessment program was performed between October and December 2010. Data from the fourth (July and December 2013) and fifth (July and December 2015) HD quality assessment programs were used in this study. These programs included adult patients (aged ≥18 years) who had undergone maintenance HD (≥3 months and ≥2 times per week).

### 2.2. Inclusion and Exclusion Criteria

**Figure 1:**
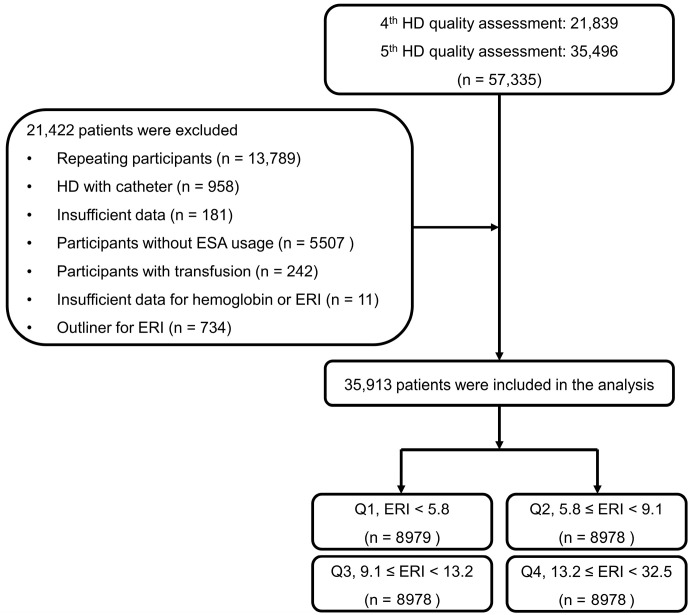
Study flowchart. Abbreviations: HD, hemodialysis, ERI, erythropoietin resistance index ((IU/week)/kg/(g/dL)); ESA, erythropoiesis-stimulating agent; Q1, first quartile; Q2, second quartile; Q3, third quartile; Q4, fourth quartile.

Among 57,335 patients included in the fourth and fifth assessments, we excluded repeat participants (*n* = 13,789), patients undergoing HD through a catheter (*n* = 958), those with insufficient data (*n* = 181), those without an ESA prescription during the assessment (*n* = 5507), those who received transfusion during the 6-month assessment period (*n* = 242), those with at least one missing hemoglobin measurement during the assessment (*n* = 11), and those identified as outliers within the 1% range on both extremes of the ERI (*n* = 734). Figure 1 shows study flowchart.

Overall, we included 35,913 patients. The institutional review board of Yeungnam University Medical Center approved this study (approval no. YUMC 2023-12-012). Informed consent was not required as patient records and information were anonymized and de-identified before analysis. Furthermore, the institutional review board of Yeungnam University Medical Center waived the informed consent requirement owing to the retrospective nature of the study. This research was conducted in adherence to ethical standards outlined in the Declaration of Helsinki.

### 2.3. Study Variables

During each HD quality assessment, data on age, sex, HD vintage (months), and vascular access type were collected. Additional clinical measures included hemoglobin (g/dL), body mass index (kg/m^2^), Kt/V_urea_, serum albumin (g/dL), serum calcium (mg/dL), serum phosphorus (mg/dL), serum creatinine (mg/dL), transferrin saturation (%), ferritin (ng/mL), and ultrafiltration volume (L/session). These data were collected monthly, with all laboratory values averaged from the monthly recordings. Kt/V_urea_ was calculated using the Daugirdas equation [12]. The doses of various ESAs were converted to a uniform unit (IU/week) using a conversion ratio from a previous study [13]. The ESA dose (IU/week) was averaged over a 6-month period. The ERI was calculated using the following equation: ERI = ESA dose (IU/week)/body weight (kg)/hemoglobin level (g/dL) [14]. Our study calculated the ERI using a mean hemoglobin value based on six monthly measurements and a mean ESA dose obtained during each 6-month HD quality assessment period. Participants were divided into quartiles based on the ERI during the 6-month assessment period: Q1, Q2, Q3, and Q4 groups.

Table S1 presents the medication codes. Medications were evaluated, including renin-angiotensin system blockers (RASBs), aspirin, clopidogrel, cinacalcet, or vitamin D analogs, including paricalcitol and statins. Medication use was defined as ≥1 prescription identified during the HD quality assessment program. Comorbidities were assessed using the Charlson comorbidity index (CCI), which includes 17 comorbidities, for 1 year before the HD quality assessment [15,16]. Additionally, myocardial infarction (MI) or congestive heart failure (CHF), atrial fibrillation, and cerebrovascular accident (CVA) were identified using ICD-10 codes.

Outcomes were evaluated from the endpoint of each HD quality assessment program to the follow-up endpoint (June 2024). All-cause mortality and CVEs were analyzed. Data on patient death were obtained from the Health Insurance Review and Assessment Service, and patients who were changed to peritoneal dialysis or received kidney transplantation without experiencing an event were censored at the time of transfer. The incidence of CVE, including MI, stroke, and revascularization, regardless of survival or death, was evaluated as previously described [17]. This outcome was evaluated for patients without these events for 6 months during the HD quality assessment program and 1 year before the program.

### 2.4. Statistical Analyses

Data were analyzed using SAS Enterprise Guide v.7.1 and R v.3.5.1. Categorical variables were presented as frequencies and percentages, while continuous variables were expressed as means with standard deviations. Statistically significant differences between categorical variables were assessed using Pearson’s χ^2^ test or Fisher’s exact test. Differences between continuous variables were examined using a one-way analysis of variance with Tukey’s post hoc test. Survival estimates were calculated using Kaplan–Meier curves and Cox regression analyses. The log-rank test was used to determine the *p*-values for comparing survival curves.

Hazard ratios (HRs) and confidence intervals were calculated using Cox regression analyses. Multivariable Cox regression analyses were adjusted for age, sex, body mass index, vascular access type, diabetes, HD vintage, CCI score, ultrafiltration volume, Kt/V_urea_, hemoglobin, serum albumin, serum creatinine, serum phosphorus, and serum calcium levels; RASB, statins, clopidogrel, or aspirin use; presence of MI or CHF, atrial fibrillation, or CVA; ESA dose per week; transferrin saturation rate; and ferritin levels. These analyses were performed using the enter mode. In the multivariable analyses, we selected covariates based on a combination of statistical significance in the univariate comparisons for baseline characteristics among groups, clinical relevance based on the prior literature, and expert clinical judgment. Specifically, variables that showed statistically significant differences between groups in the baseline characteristics were considered for inclusion, as these characteristics could represent potential confounders. Additionally, even if some variables were not statistically significant in the univariable analyses (using clopidogrel and CVA), any variable deemed clinically important or previously reported in the literature as associated with patient outcomes was still included. This approach reflects our intention to build a statistically sound and clinically meaningful model. Importantly, our study included a sufficiently large number of patients, which allowed the adjustment for a comprehensive set of covariates without compromising the statistical power or stability of the model. We believe this strengthens the validity of our findings and reduces the risk of residual confounding. Most baseline variables in our study population were closely linked with clinical outcomes. Therefore, we aimed to adjust for these variables to minimize bias and improve the robustness of our results. A restricted cubic spline curve was employed to evaluate non-linear relationships between ERI and patient death or CVE incidence adjusted for covariates. In addition, we performed ordinal logistic regression analyses to assess the factors associated with an increase in the ERI quartiles. A *p* < 0.05 indicated statistical significance.

Significant differences in baseline characteristics were observed among the four groups. We applied propensity score weighting to attenuate potential bias, ensuring the validity of our analysis. We constructed a balanced cohort for the four groups using generalized boosted models, adjusting for age, sex, body mass index, diabetes, vascular access type, CCI score, HD vintage, ultrafiltration volume, Kt/V_urea_, hemoglobin, creatinine, phosphorus, albumin, calcium, transferrin saturation, ferritin, and ERI levels; ESA dose; aspirin, statin, RASB, or clopidogrel use; and the presence of MI or CHF, atrial fibrillation, and CVA. Propensity scores were used to calculate inverse probability treatment weights. The balanced cohort was defined as a sample with weights assigned to each case, with continuous variables presented as means and standard errors. *p* values were tested using a general linear model with a complex survey design, incorporating sample weights.

## 3. Results

### 3.1. Clinical Characteristics

The Q1, Q2, Q3, and Q4 groups comprised 8979, 8978, 8978, and 8978 patients, respectively (Table 1).

The ERI in Q1, Q2, Q3, and Q4 groups was 3.6 ± 1.5, 7.5 ± 0.9, 11.0 ± 1.2, and 18.0 ± 4.2 (IU/week)/kg/(g/dL), respectively. Patients in the Q4 group were older compared to the other groups. Additionally, this group had a higher HD vintage, Kt/V_urea_, CCI scores, and ferritin levels, as well as a higher proportion RASB use and heart diseases than the other groups. However, the Q4 had a lower proportion of diabetes and transferrin saturation, hemoglobin, albumin, calcium, phosphorus, and creatinine levels than the other groups. The Q1 group had a higher proportion of males, aspirin and statin use, and autologous arteriovenous fistula, as well as a higher body mass index, than the other groups. We compared the usage rates of vitamin D analogs or cinacalcet, commonly used in treating hyperparathyroidism. The Q1, Q2, Q3, and Q4 numbers were 4590 (51.1%), 4566 (50.8%), 4653 (51.8%), and 4605 (51.3%), respectively (*p* = 0.633).

### 3.2. All-Cause Mortality and CVE According to Groups

The follow-up for Q1, Q2, Q3, and Q4 groups lasted 76 ± 38, 75 ± 39, 75 ± 39, and 70 ± 40 months, respectively. At the end of the follow-up, patient outcomes respectively included survival, death, transfer to peritoneal dialysis, or kidney transplantation: 3254 (36.2%), 4702 (52.4%), 32 (0.4%), and 991 (11.0%) for the Q1 group; 3185 (35.5%), 4790 (53.4%), 35 (0.4%), and 968 (10.8%) for the Q2 group; 3058 (34.1%), 4919 (54.8%), 41 (0.5%), and 960 (10.7%) for the Q3 group; and 2700 (30.1%), 5483 (61.1%), 40 (0.4%), and 755 (8.4%) for the Q4 group (*p* < 0.001).

**Figure 2:**
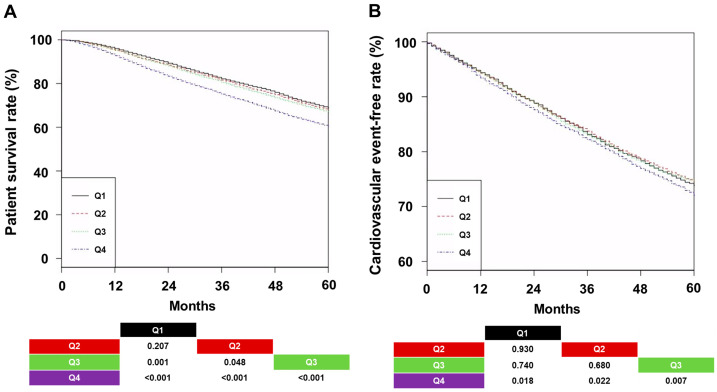
Kaplan–Meier curves by quartiles of the erythropoietin resistance index. (**A**) Patient survival and (**B**) cardiovascular event–free rates. *p*-values for pairwise comparisons with log-rank tests were added to the bottom of the graph. Abbreviations: Q1, first quartile; Q2, second quartile; Q3, third quartile; Q4, fourth quartile.

The 5-year survival rates were 68.8% (Q1), 67.8% (Q2), 66.9% (Q3), and 60.2% (Q4) (Figure 2A; *p* < 0.001).

The 5-year CVE-free rates were 73.8% (Q1), 74.4% (Q2), 74.4% (Q3), and 72.1% (Q4) (Figure 2B; *p* = 0.030). The Q4 group had the shortest patient survival and the lowest CVE-free rate compared to the other groups. Univariable analysis showed that the HR for all-cause mortality was higher in Q3 and Q4 groups than in Q1 and Q2 groups (Table 2).

**Figure 3:**
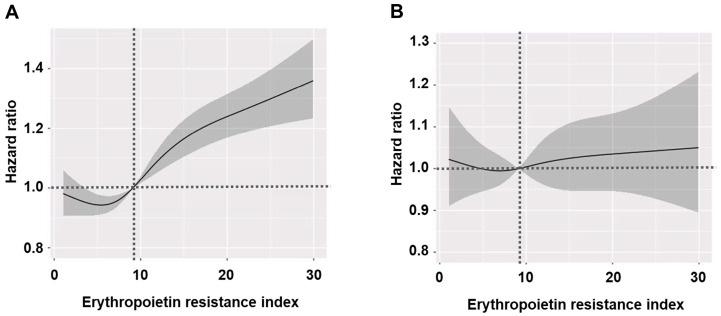
Spline curves illustrating hazard ratios and 95% confidence intervals for clinical outcomes according to the erythropoietin resistance index. (**A**) All-cause mortality. (**B**) Cardiovascular events. The solid line shows the hazard ratio estimated from the multivariable model with a reference point at 9.1 (IU/week)/kg/(g/dL) of the erythropoietin resistance index, the dashed line represents the hazard ratio of 1.0 (reference), and the gray shaded area indicates the 95% confidence interval.

Multivariable analysis showed the same trends as the univariable analysis. Additionally, a spline curve using the multivariable model indicated that the increased ERI, based on a median of 9.1 (IU/week)/kg/(g/dL), was linked to all-cause mortality (Figure 3).

However, CVE was not associated with ERI quartiles in univariable and multivariable Cox regression analyses.

### 3.3. Subgroup Analysis

We conducted subgroup analyses based on sex, age (<65 or ≥65 years), CCI score (<7 or ≥7, defined as the mean CCI score), hemoglobin level (<10 or ≥10 g/dL, defined as the mean hemoglobin level), ESA dose (<5660 or ≥5660 IU/week, defined as the mean ESA dose), and HD vintage (<40 or ≥40 months, defined as the mean HD vintage) to determine the specific groups in which the effects of ERI were more pronounced. The hazard effect of the Q4 group was similar in most subgroups (Table 3).

However, a hazard effect for CVE in the Q4 group was not observed in most subgroups (Table S2). 

### 3.4. Analyses Using the Balanced Cohort

Significant differences were observed in all variables among the four groups in the original cohort. Then, we conducted analysis using the cohort after propensity score weighting. Balance among the three groups was assessed by calculating the maximum pairwise absolute standardized mean differences (ASMDs) of the covariates before and after balancing (Figure S1). After weighting, the maximum ASMDs and baseline characteristic differences were reduced for all covariates. The numbers of patients in Q1, Q2, Q3, and Q4 groups using the weighted cohort were 34,043, 34,346, 34,440, and 36,142, respectively. Table S3 shows the baseline characteristics after weighting, with attenuated characteristic differences.

The 5-year survival rates were 66.9% (Q1), 66.9% (Q2), 66.5% (Q3), and 63.7% (Q4) (Figure S2A, *p* < 0.001). The 5-year CVE-free rates were 73.1% (Q1), 74.2% (Q2), 74.2% (Q3), and 72.9% (Q4) (Figure S2B, *p* = 0.603). The Q4 group had the shortest patient survival rate among the four groups. Univariable and multivariable analyses of all-cause mortality indicated that the HR in the Q4 group was the highest among the four groups (Table S4).

### 3.5. Clinical Factors Associated with ERI

We performed a multivariable ordinal logistic regression analysis using multiple variables to identify factors associated with increased ERI quartiles (Table S5). In this analysis, increased ages, CCI, ultrafiltration volume, serum creatinine, female sex, use of arteriovenous graft, use of RASB, and the presence of MI or CHF tended to be associated with higher ERI quartiles. In contrast, higher body mass index, Kt/V_urea_, hemoglobin, albumin, and transferrin saturation rate, along with statin use and the presence of CVA or diabetes, tended to be associated with lower ERI quartiles. Overall, ERI quartiles tended to be higher in patients with underlying comorbidities and those with poor dialysis adequacy or higher ultrafiltration volume. Although definitive conclusions cannot be drawn, the trend of an increased ERI following RASB use and a decreased ERI after statin use is consistent with previous studies [18,19]. Notably, the presence of CVA and diabetes showed a tendency toward a lower ERI, which may be influenced by the higher proportion of female sex, younger age, and the effects of various medications used in the Q1 group.

## 4. Discussion

Our study explored the association between ESA responsiveness, measured using the ERI, and clinical outcomes in a large cohort of 35,913 HD patients. We found that a high ERI was associated with all-cause mortality, although CVE rates did not differ notably between ERI groups. The Q4 group increased the risk of all-cause mortality by 24% in the Q1 group, 22% in the Q2 group, and 14% in the Q3 group. Subgroup analysis revealed that in most subgroups, the all-cause mortality was significantly higher in those with a high ERI than in those with a low ERI. Further analysis using the balanced cohort, which attenuated baseline characteristic differences, confirmed that the high mortality in those with the high ERI was maintained.

ESAs bind to the erythropoietin (EPO) receptor on erythrocyte progenitor cells, promoting their anti-apoptotic activity, differentiation, and proliferation. This ultimately leads to red blood cell production. However, using ESAs, particularly at high doses, can sometimes induce rapid erythropoiesis exceeding the adaptive capacity of the cardiovascular system, resulting in hypertension and heart failure [20]. Moreover, ESAs are implicated in angiogenesis and pleiotropic effects mediated through the EPO receptor, raising concerns about potential cancer progression [21]. Additionally, despite limited clinical data, various studies suggested a possible association between ESA use and the worsening of diabetic retinopathy [22]. Beyond these concerns, ESAs are also linked to thromboembolic risk, including vascular access thrombosis, which may ultimately contribute to reduced patient survival. Therefore, while ESAs are essential for anemia correction, using the lowest effective dose is recommended to minimize potential adverse effects. The dosage or resistance to ESA therapy required to maintain an appropriate hemoglobin level can be quantified using a simple metric known as the ERI. While the ERI formula used in this study follows the standard definition proposed by Fishbane and Bern [14], recent studies have highlighted the influence of inflammation, dialysis modality, and nutritional status on ERI values [23,24]. These findings suggest the ERI may reflect broader clinical conditions beyond ESA responsiveness alone.

Many studies examining the association between the ERI and mortality in maintenance HD patients were conducted at the single-center or cohort level rather than as population-sized studies. Notably, among the Asian population, research has primarily focused on Chinese and Japanese patients. In studies targeting the Chinese population, Zao et al. analyzed data from 1270 Chinese patients enrolled in the Dialysis Outcomes and Practice Patterns Study (DOPPS), demonstrating an association between the ERI and all-cause mortality [8]. Pan et al. evaluated 824 HD patients from 16 centers in China and found that a high ERI quartile was associated with all-cause mortality; the same results were obtained from analyses using a propensity score matching cohort between high- or low-ERI groups [9]. A single-center study in China also showed a positive association between a high ERI and cardiovascular-specific mortality or clinical variables such as C-reactive protein or ferritin [25]. Similarly, several studies using Japanese data were conducted, including those based on single-center analyses or utilizing DOPPS data. In particular, a study analyzing 2104 Japanese patients from the DOPPS also confirmed the association between these two variables [26]. Three Japanese studies that applied a single-center design showed an association between the ESA dose or ERI and all-cause mortality [24,27,28]. A post-hoc analysis using data from a Q-cohort also showed an association between a high ERI tertile and all-cause mortality [29]. Furthermore, a study utilizing data from a prospective cohort in Korea analyzed 1594 HD patients, showing an association between ERI and all-cause mortality [30]. Beyond Asia, the RISCAVID study, a study using regional data from Italy, yielded similar findings [31].

Although previous studies have examined the relationship between ERI and clinical outcomes using data collected through single-center, multicenter, or retrospective cohort designs, the sample size is the most significant difference between those studies and this study. To calculate the ERI, it is essential to have access to laboratory data, including hemoglobin levels, and data confirming the ESA dose, which can be obtained from claims or prescription records. Studies that rely solely on claims data may have the advantage of utilizing large sample sizes and can provide information on ESA prescriptions. However, such studies face limitations in collecting laboratory data, making direct ERI calculations challenging. Conversely, single-center or multicenter studies can collect more comprehensive laboratory and clinical data, allowing the inclusion of various confounding factors. However, these studies often struggle to achieve a nationwide sample size. Thus, our study represents a compromise between these two approaches. The baseline dataset in our study was not originally collected for research purposes but as part of a national quality improvement initiative for all registered HD centers in South Korea. As such, this dataset included only the minimal laboratory data necessary for quality monitoring. Based on this foundational dataset, we were able to anonymize patient information and link these data with claims data to calculate the ERI and examine any association with clinical outcomes. Nevertheless, due to the nature of a nationwide program, our study also has limitations in collecting comprehensive laboratory or clinical data compared to other studies.

Our study shows the association between many clinical factors and the ERI. These data reveal that the ERI can be a simple marker for underlying diseases associated with poor outcomes rather than a cause of death. Nevertheless, considering the results from multivariable analyses adjusted for these variables and findings from previous studies on the independent effect of the ERI, some degree of association between the ERI and mortality likely exists. Therefore, further studies are needed to clarify the independent impact of each variable on the ERI.

Iron status, hyperparathyroidism, and malnutrition–inflammation complex syndrome are important confounding factors associated with the ERI. Our dataset included transferrin saturation rate, ferritin, body mass index, and serum albumin, the latter two of which are well-known nutritional indicators, while serum albumin is also a negative reactive protein. Hence, we evaluated the association between the ERI and these variables. Increased serum albumin, transferrin saturation rate, and body mass index were associated with high ERI quartiles. We have performed multivariable analyses that included these covariates. Furthermore, we compared the usage rates of vitamin D analogs or cinacalcet, commonly used in treating hyperparathyroidism; no significant differences were noted in the usage rates of these medications. Although our dataset did not include the direct indicators for hyperparathyroidism, such as intact parathyroid hormone or alkaline phosphatase, it may be possible that hyperparathyroidism did not serve as a confounding factor influencing the association between the ERI and all-cause mortality.

Recently, there has been an increasing number of analyses using population-based data derived from claim databases. However, studies directly investigating the relationship between actual ERI values and mortality remain scarce. This limitation is likely due to the lack of laboratory data. The calculation of the ERI requires information on the administered ESA dose, body weight, and hemoglobin level. While ESA dose can be easily obtained from claim data, body weight and hemoglobin levels are difficult to verify solely from these sources. In contrast, our study utilized a dataset derived from the HD quality assessment program, which collected monthly hemoglobin levels over a 6-month period from all participating centers as part of an HD center grading initiative. We were able to accurately calculate the ERI and conduct outcome analyses by integrating these laboratory data with claim data, making our study feasible.

In our study, the CVE risk was not associated with the ERI, regardless of whether it was analyzed as a categorical variable or a continuous variable. This finding may be linked to the limitations of our study. Our analysis identified CVEs using ICD-10 codes without additional validation through medical chart reviews or patient interviews, or the presence or absence of specific CVE-related procedures. In particular, the reliance on ICD-10 codes might have influenced our findings, as these codes are often assigned independently of the actual presence of disease. This might have affected the observed association between the ERI and CVEs in our study. Another possible explanation is that the ERI may not be a strong enough independent factor to significantly influence the CVE risk, especially when compared to well-established classical risk factors. In fact, our data showed a trend toward the increased CVE risk in the Q4 group, although this difference was not statistically significant. Furthermore, in contrast to CVE findings, the ERI demonstrated a significant association with all-cause mortality, representing a final outcome influenced by various complications, including CVEs. This discrepancy may be attributed to the inaccuracy of CVE diagnosis, the potential indirect effects of ERI-related factors, or other hazardous effects of ERI beyond CVEs.

The recent KDIGO guidelines identify iron deficiency and inflammation as the most common causes of ESA hyporesponsiveness [32]. Meanwhile, evaluating iron status is recommended to differentiate between absolute and functional iron deficiency; for absolute iron deficiency, active iron supplementation—either intravenous or oral—should be administered. Inflammation is also a significant contributor to ESA hyporesponsiveness. Inflammatory cytokines can induce hyporesponsiveness either directly or indirectly through hepcidin. Therefore, controlling underlying sources of inflammation, such as infections, should be prioritized. Although evidence supporting the efficacy of anti-inflammatory pharmacologic treatments in improving ESA hyporesponsiveness remains limited, recent studies have reported that using medium cut-off dialyzers, which remove inflammatory cytokines, can improve ESA responsiveness [23]. Other interventions, such as biocompatible membranes, ultrapure dialysate, and short daily dialysis, have enhanced ESA responsiveness by reducing inflammation [33,34,35]. Furthermore, although evidence remains limited, recent reports suggest that continuous erythropoietin receptor activators may reduce hepcidin levels [36]. After weighing the potential risks and benefits, the KDIGO guidelines also indicate that hypoxia-inducible factor prolyl hydroxylase inhibitors may be considered [32].

Our study has some limitations. First, it was retrospective. Additionally, the original cohort exhibited significant differences in almost all baseline characteristics, making determining the independent prognostic impact of the ERI difficult. Therefore, we performed two sets of analyses, which included multivariable analyses, subgroup analyses, and analyses using a balanced cohort weighted by propensity score. We attempted to adjust for most variables that could affect the prognosis; in particular, we created a balanced cohort using propensity score weighting to reduce most of the unmatched baseline characteristics substantially. Furthermore, we performed multivariable analyses within this balanced cohort, and the results for all-cause mortality were consistent with those obtained from the original cohort. Nevertheless, the possibility of residual bias cannot be completely excluded, and well-designed randomized controlled trials are warranted to confirm the independent effect. Second, specific data related to high ERI, such as hyperparathyroidism or inflammation, were unavailable. These unmeasured variables are known to influence ESA responsiveness and could have acted as confounders. Although we adjusted for several clinical parameters, including nutritional and iron status, the lack of direct measurements for inflammation and mineral metabolism is a limitation. Third, omitting individuals with missing hemoglobin values or extreme outliers—necessary for an accurate ERI calculation—may have introduced selection bias by excluding patients with unstable clinical conditions. Although this approach improved data integrity, it may have led to an underrepresentation of certain patient subgroups. Nevertheless, the number of patients with missing hemoglobin values and outliers was 11 and 734, respectively, and the small number of patients may not largely influence our results. Furthermore, identifying which complications, such as CVEs, infection, or bleeding, primarily contributed to mortality would be highly valuable. However, while we could confirm death occurrences, we lacked the necessary data to analyze the specific causes of death. Several strategies could be employed in future studies to minimize bias further. A prospective cohort design would allow for systematic data collection of key confounders such as inflammatory markers and intact parathyroid hormone levels. Using multiple imputation techniques to address missing values and sensitivity analyses to evaluate the robustness of these findings would also help reduce the impact of selection and information bias. Additionally, collecting detailed cause-of-death data and considering matched case-control approaches may enhance causal inference regarding the prognostic implications of the ERI.

In conclusion, our population-based cohort study reveals an association between the ERI and all-cause mortality in HD patients. This highlights the need for regular ERI monitoring and the importance of actively identifying and correcting the underlying causes in patients with high ERI to reduce it to an appropriate level. However, further research is needed to confirm this association more definitively due to the various limitations of this study.

## Figures and Tables

**Table 1:** Baseline characteristics.

	Q1 (*n* = 8979)	Q2 (*n* = 8978)	Q3 (*n* = 8978)	Q4 (*n* = 8978)	*p*-Value
Age (years)	59.6 ± 12.7	60.5 ± 12.8 ^a^	60.7 ± 12.8 ^a^	61.8 ± 12.7 ^abc^	<0.001
Sex (male, %)	6190 (68.9%)	5600 (62.4%)	4867 (54.2%)	4175 (46.5%)	<0.001
HD vintage (months)	63 ± 66	55 ± 58 ^a^	59 ± 60 ^ab^	66 ± 65 ^abc^	<0.001
BMI (kg/m^2^)	23.3 ± 3.5	22.7 ± 3.2 ^a^	22.1 ± 3.2 ^ab^	21.2 ± 3.0 ^abc^	<0.001
Diabetes (%)	4125 (45.9%)	4220 (47.0%)	3875 (43.2%)	3659 (40.8%)	<0.001
CCI score	6.85 ± 2.71	6.82 ± 2.67	6.78 ± 2.73	6.95 ± 2.75 ^bc^	<0.001
Arteriovenous fistula (%)	7758 (86.4%)	7706 (85.8%)	7681 (85.6%)	7468 (83.2%)	<0.001
Kt/V_urea_	1.48 ± 0.26	1.51 ± 0.27 ^a^	1.54 ± 0.26 ^ab^	1.58 ± 0.28 ^abc^	<0.001
UFV (L/session)	2.28 ± 1.03	2.25 ± 0.95	2.30 ± 0.93 ^b^	2.32 ± 0.93 ^ab^	<0.001
Hemoglobin (g/dL)	11.0 ± 0.7	10.7 ± 0.6 ^a^	10.6 ± 0.6 ^ab^	10.3 ± 0.7 ^abc^	<0.001
Serum albumin (g/dL)	4.01 ± 0.33	4.00 ± 0.33	3.99 ± 0.34 ^ab^	3.93 ± 0.36 ^abc^	<0.001
Serum phosphorus (mg/dL)	4.94 ± 1.30	4.97 ± 1.29	4.98 ± 1.35	4.88 ± 1.38 ^abc^	<0.001
Serum calcium (mg/dL)	8.93 ± 0.80	8.91 ± 0.82	8.93 ± 0.84	8.90 ± 0.86 ^a^	0.011
Serum creatinine (mg/dL)	9.59 ± 2.90	9.61 ± 2.75	9.60 ± 2.61	9.18 ± 2.46 ^abc^	<0.001
Use of RASB (%)	5473 (61.0%)	6329 (70.5%)	6616 (73.7%)	6697 (74.6%)	<0.001
Use of aspirin (%)	1234 (13.7%)	1182 (13.2%)	1119 (12.5%)	1107 (12.3%)	0.016
Use of clopidogrel (%)	717 (8.0%)	738 (8.2%)	679 (7.6%)	671 (7.5%)	0.199
Use of statins (%)	4054 (45.1%)	3898 (43.4%)	3658 (40.7%)	3399 (37.9%)	<0.001
MI or CHF (%)	3485 (38.8%)	3448 (38.4%)	3548 (39.5%)	3767 (42.0%)	<0.001
Atrial fibrillation (%)	672 (7.5%)	596 (6.6%)	648 (7.2%)	776 (8.6%)	<0.001
CVA (%)	2398 (26.7%)	2386 (26.6%)	2286 (25.5%)	2374 (26.4%)	0.216
Transferrin saturation (%)	35.2 ± 28.7	35.5 ± 27.0	34.4 ± 25.2 ^b^	32.9 ± 28.1 ^abc^	<0.001
Ferritin (ng/mL)	254 ± 257	266 ± 250 ^a^	270 ± 255 ^a^	281 ± 287 ^abc^	<0.001
ESA dose (IU/week)	2465 ± 1141	4895 ± 1006 ^a^	6791 ± 1331 ^ab^	10,053 ± 2551 ^abc^	<0.001

Data are expressed as means ± standard deviation for continuous variables and numbers (percentages) for categorical variables. *p*-values were tested using one-way analysis of variance, followed by the Tukey post hoc test. Pearson’s χ^2^ test was performed for categorical variables. ^a^ *p* < 0.05 vs. Q1, ^b^ *p* < 0.05 vs. Q2, and ^c^ *p* < 0.05 vs. Q3. Abbreviations: BMI, body mass index; CCI, Charlson Comorbidity Index; CHF, congestive heart failure; CVA, cerebrovascular accidents; HD, hemodialysis; ESA, erythropoiesis-stimulating agent; Kt/V_urea_, dialysis adequacy; MI, myocardial infarction; RASB, renin-angiotensin system blocker; UFV, ultrafiltration volume; Q1, first quartile; Q2, second quartile; Q3, third quartile; Q4, fourth quartile.

**Table 2:** Erythropoietin resistance index and HR of all-cause mortality or CVE.

	Univariable		Multivariable	
	HR (95% CI)	*p*-Value	HR (95% CI)	*p*-Value
All-Cause Mortality				
Reference: Q1				
Q2	1.03 (0.99–1.07)	0.206	1.02 (0.97–1.07)	0.405
Q3	1.07 (1.03–1.11)	<0.001	1.09 (1.02–1.17)	0.014
Q4	1.28 (1.23–1.33)	<0.001	1.24 (1.15–1.35)	<0.001
Reference: Q2				
Q3	1.04 (1.00–1.09)	0.041	1.07 (1.01–1.13)	0.025
Q4	1.25 (1.20–1.30)	<0.001	1.22 (1.14–1.30)	<0.001
Reference: Q3				
Q4	1.20 (1.15–1.25)	<0.001	1.14 (1.09–1.19)	<0.001
CVE				
Reference: Q1				
Q2	1.00 (0.94–1.06)	0.931	0.99 (0.92–1.06)	0.680
Q3	0.99 (0.93–1.05)	0.743	0.98 (0.88–1.08)	0.644
Q4	1.07 (1.01–1.14)	0.018	1.03 (0.91–1.16)	0.635
Reference: Q2				
Q3	0.99 (0.93–1.05)	0.678	0.99 (0.91–1.08)	0.824
Q4	1.07 (1.01–1.14)	0.022	1.05 (0.94–1.16)	0.397
Reference: Q3				
Q4	1.09 (1.02–1.15)	0.007	1.06 (0.98–1.13)	0.127

Multivariate analysis was adjusted for age; sex; body mass index; vascular access type; diabetes; hemodialysis vintage; Charlson Comorbidity Index score; ultrafiltration volume; Kt/V_urea_; hemoglobin, serum albumin, serum creatinine, serum phosphorus, and serum calcium levels; renin-angiotensin system blockers, statin, clopidogrel, or aspirin use; presence of myocardial infarction or congestive heart failure, atrial fibrillation, or cerebrovascular accidents; erythropoiesis-stimulating agent dose per week; transferrin saturation rate; and ferritin levels. Abbreviations: CI, confidence interval; CVE, cardiovascular event; HR, hazard ratio; Kt/V_urea_, dialysis adequacy; Q1, first quartile; Q2, second quartile; Q3, third quartile; Q4, fourth quartile.

**Table 3:** Erythropoietin resistance index and HR of all-cause mortality according to subgroups.

	Univariable		Multivariable			Univariable		Multivariable	
	HR (95% CI)	*p*	HR (95% CI)	*p*		HR (95% CI)	*p*	HR (95% CI)	*p*
Males					Females				
Reference: Q1									
Q2	1.07 (1.01–1.12)	0.012	1.00 (0.95–1.06)	0.931		0.98 (0.92–1.06)	0.628	1.02 (0.94–1.10)	0.623
Q3	1.13 (1.07–1.18)	<0.001	1.03 (0.93–1.13)	0.601		1.04 (0.98–1.12)	0.203	1.14 (1.03–1.26)	0.013
Q4	1.45 (1.38–1.52)	<0.001	1.18 (1.07–1.32)	0.002		1.21 (1.14–1.30)	<0.001	1.29 (1.14–1.47)	<0.001
Reference: Q2									
Q3	1.06 (1.00–1.11)	0.033	1.02 (0.95–1.11)	0.565		1.06 (0.99–1.13)	0.061	1.12 (1.02–1.22)	0.018
Q4	1.36 (1.29–1.43)	<0.001	1.18 (1.08–1.29)	<0.001		1.24 (1.16–1.31)	<0.001	1.27 (1.13–1.42)	<0.001
Reference: Q3									
Q4	1.29 (1.22–1.36)	<0.001	1.15 (1.09–1.23)	<0.001		1.16 (1.10–1.23)	<0.001	1.14 (1.06–1.22)	<0.001
<65 years old					≥65 years old				
Reference: Q1									
Q2	0.98 (0.92–1.05)	0.556	0.94 (0.88–1.02)	0.128		1.01 (0.96–1.06)	0.765	1.00 (0.94–1.06)	0.976
Q3	1.07 (1.00–1.14)	0.042	1.04 (0.93–1.16)	0.537		0.99 (0.95–1.05)	0.869	1.01 (0.93–1.11)	0.776
Q4	1.26 (1.18–1.34)	<0.001	1.14 (0.99–1.29)	0.051		1.20 (1.14–1.26)	<0.001	1.14 (1.03–1.27)	0.014
Reference: Q2									
Q3	1.09 (1.02–1.16)	0.009	1.10 (1.01–1.20)	0.048		0.99 (0.94–1.04)	0.636	1.01 (0.94–1.09)	0.757
Q4	1.28 (1.20–1.36)	<0.001	1.20 (1.08–1.34)	<0.001		1.19 (1.13–1.25)	<0.001	1.14 (1.04–1.25)	0.004
Reference: Q3									
Q4	1.18 (1.11–1.25)	<0.001	1.10 (1.02–1.18)	0.011		1.20 (1.14–1.26)	<0.001	1.13 (1.06–1.19)	<0.001
Low CCI (<7)					High CCI (≥7)				
Reference: Q1									
Q2	0.98 (0.92–1.05)	0.655	0.92 (0.85–0.99)	0.040		1.05 (1.00–1.11)	0.041	1.08 (1.01–1.14)	0.015
Q3	1.07 (1.00–1.14)	0.037	1.04 (0.93–1.17)	0.464		1.09 (1.03–1.14)	0.001	1.11 (1.02–1.22)	0.017
Q4	1.29 (1.21–1.37)	<0.001	1.14 (0.99–1.31)	0.051		1.29 (1.23–1.35)	<0.001	1.28 (1.16–1.42)	<0.001
Reference: Q2									
Q3	1.09 (1.02–1.16)	0.011	1.13 (1.03–1.25)	0.013		1.03 (0.98–1.09)	0.220	1.03 (0.96–1.11)	0.369
Q4	1.02 (0.95–1.09)	<0.001	1.24 (1.10–1.39)	<0.001		1.22 (1.17–1.28)	<0.001	1.19 (1.10–1.30)	<0.001
Reference: Q3									
Q4	1.20 (1.13–1.28)	<0.001	1.10 (1.02–1.18)	0.017		1.19 (1.13–1.24)	<0.001	1.15 (1.09–1.22)	<0.001
Low Hb (<10)					High Hb (≥10)				
Reference: Q1									
Q2	1.03 (0.88–1.20)	0.706	0.97 (0.82–1.16)	0.758		1.02 (0.98–1.07)	0.270	1.03 (0.98–1.08)	0.217
Q3	1.00 (0.87–1.14)	0.952	0.94 (0.76–1.15)	0.531		1.07 (1.03–1.12)	0.001	1.12 (1.04–1.21)	0.002
Q4	1.33 (1.18–1.51)	<0.001	1.12 (0.89–1.40)	0.353		1.22 (1.16–1.27)	<0.001	1.25 (1.15–1.37)	<0.001
Reference: Q2									
Q3	0.97 (0.85–1.10)	0.600	0.96 (0.81–1.15)	0.672		1.05 (1.00–1.09)	0.033	1.09 (1.02–1.16)	0.007
Q4	1.29 (1.16–1.44)	<0.001	1.15 (0.94–1.40)	0.181		1.19 (1.14–1.24)	<0.001	1.22 (1.13–1.31)	<0.001
Reference: Q3									
Q4	1.34 (1.23–1.46)	<0.001	1.19 (1.07–1.33)	0.002		1.13 (1.08–1.18)	<0.001	1.12 (1.06–1.17)	<0.001
Low ESA dose					High ESA dose				
Reference: Q1									
Q2	1.04 (0.99–1.08)	0.108	1.09 (1.01–1.18)	0.026		1.74 (0.83–3.66)	0.145	0.91 (0.43–1.92)	0.803
Q3	1.11 (1.04–1.19)	0.003	1.26 (1.12–1.43)	<0.001		1.86 (0.89–3.91)	0.100	0.92 (0.44–1.96)	0.837
Q4	2.41 (1.77–3.28)	<0.001	2.37 (1.65–3.40)	<0.001		2.25 (1.07–4.71)	0.033	1.05 (0.49–2.24)	0.894
Reference: Q2									
Q3	1.07 (0.99–1.15)	0.057	1.16 (1.06–1.27)	0.001		1.07 (1.00–1.15)	0.048	1.02 (0.94–1.10)	0.692
Q4	2.33 (1.71–3.17)	<0.001	2.17 (1.53–3.07)	<0.001		1.29 (1.21–1.38)	<0.001	1.16 (1.04–1.29)	0.006
Reference: Q3									
Q4	2.17 (1.59–2.97)	<0.001	1.87 (1.32–2.65)	<0.001		1.21 (1.16–1.26)	<0.001	1.14 (1.07–1.21)	<0.001
Short HDV (<40 M)					Long HDV (≥40 M)				
Reference: Q1									
Q2	1.04 (0.98–1.10)	0.181	1.07 (0.99–1.14)	0.056		1.02 (0.96–1.08)	0.541	0.98 (0.92–1.05)	0.656
Q3	1.11 (1.05–1.17)	<0.001	1.13 (1.02–1.24)	0.016		1.03 (0.98–1.09)	0.275	1.06 (0.96–1.17)	0.220
Q4	1.36 (1.29–1.44)	<0.001	1.34 (1.19–1.50)	<0.001		1.21 (1.14–1.28)	<0.001	1.15 (1.03–1.30)	0.014
Reference: Q2									
Q3	1.06 (1.01–1.13)	0.027	1.06 (0.98–1.15)	0.171		1.01 (0.96–1.07)	0.646	1.08 (0.99–1.18)	0.074
Q4	1.31 (1.24–1.39)	<0.001	1.25 (1.14–1.38)	<0.001		1.19 (1.12–1.25)	<0.001	1.17 (1.06–1.29)	0.002
Reference: Q3									
Q4	1.23 (1.17–1.30)	<0.001	1.18 (1.11–1.26)	<0.001		1.17 (1.11–1.23)	<0.001	1.09 (1.02–1.16)	0.011

Multivariate analysis was adjusted for age; sex; body mass index; vascular access type; diabetes; hemodialysis vintage; CCI score; ultrafiltration volume; Kt/V_urea_; hemoglobin, serum albumin, serum creatinine, serum phosphorus, and serum calcium levels; renin-angiotensin system blockers, statin, clopidogrel, or aspirin use; presence of myocardial infarction or congestive heart failure, atrial fibrillation, or cerebrovascular accidents; ESA dose per week; erythropoietin resistance index; transferrin saturation rate; and ferritin levels. Abbreviations: CCI, Charlson Comorbidity Index; CI, confidence interval; ESA, erythropoiesis stimulating agent; HR, hazard ratio; Kt/V_urea_, dialysis adequacy; low CCI (<7), the subset with CCI scores < 7; high CCI (≥7), the subset with CCI scores ≥ 7; low Hb (<10), the subgroup with hemoglobin levels <10 g/dL; high Hb (≥10), the subgroup with hemoglobin levels ≥ 10 g/dL; low ESA dose, the subgroup with mean ESA doses < 5660 IU/week; high ESA dose, the subgroup with mean ESA doses ≥ 5660 IU/week; short HDV (<40 M), the subgroup with dialysis durations < 40 months; long HDV (≥40 M), the subgroup with dialysis durations ≥ 40 months; Q1, first quartile; Q2, second quartile; Q3, third quartile; Q4, fourth quartile.

## Data Availability

The raw data were generated at the Health Insurance Review and Assessment Service. The database can be requested from the Health Insurance Review and Assessment Service by sending a study proposal including the purpose of the study, study design, and duration of analysis through an e-mail (turtle52@hira.or.kr) or at the web site (https://www.hira.or.kr, accessed on 16 April 2025). The authors cannot distribute the data without permission.

## Supplementary Materials

The following supporting information can be downloaded at https://www.mdpi.com/article/10.3390/jcm14082812/s1: Table S1. Medication types and Health Insurance Review and Assessment Service codes; Table S2. Erythropoietin resistance index and HR of CVEs according to subgroups; Table S3. Baseline characteristics using a balanced cohort; Table S4. Erythropoietin resistance index and HR using a balanced cohort; Table S5. Factors associated with an increase in the ERI quartiles; Figure S1. Balance tests; Figure S2. Kaplan–Meier curves by quartiles of erythropoietin resistance index using a balanced cohort.
