# Supplementary material for: The Association Between Erythropoiesis Resistance Index and Clinical Outcomes in Hemodialysis Patients: A Nationwide Study
Source: J Clin Med. 2025 Apr 18;14(8):2812. doi: 10.3390/jcm14082812 (PMC12028047; doi:10.3390/jcm14082812)
Supplement: Supplementary file 1 [file jcm-14-02812-s001.zip › jcm-3557650-supplementary.pdf]

Table S1. Medication types and Health Insurance Review and Assessment Service codes

Table S2. Erythropoietin resistance index and HR of CVEs according to subgroups

Table S3. Baseline characteristics using a balanced cohort

Table S4. Erythropoietin resistance index and HR using a balanced cohort

Table S5. Factors associated with an increase in ERI quartiles

Figure S1. Balance tests.

Figure S2. Kaplan-Meier curves by quartiles of erythropoietin resistance index using a balanced cohort

Table S1. Medication types and Health Insurance Review and Assessment Service codes

**Medications**	**Codes**
**Alacepril**	104201, 104202
**Benazepril**	114701
**Captopril**	122901, 122902, 122903
**Cilazapril**	133001, 133002, 133003
**Enalapril**	151601, 151603
**Fosinopril**	163501, 163502
**Imidapril**	173401, 173402
**Moexipril**	196801, 196802
**Lisinopril**	184501
**Perindopril**	211301, 211302, 501601, 501602
**Quinapril**	221901,
**Ramipril**	222401, 222402, 222404
**Zofenopril**	510401, 510402, 510403
**Temocapril**	235002
**Delapril**	140901, 140902
**Captopril + Hydrochlorothiazide**	262200, 262300
**Enalapril + Hydrochlorothiazide**	440300, 453700, 453600
**Ramipril + Felodipine**	447100, 447200
**Ramipril + Hydrochlorothiazide**	448600, 448700
**Perindopril + indapamide**	556200
**Lisinopril + Hydrochlorothiazide**	499200, 499300
**Moexipril + Hydrochlorothiazide**	440800, 497900
**Enalapril + nitrendipine**	466000
**Candesartan**	122601, 122602, 122603
**Irbesartan**	177301, 177303
**Losartan**	185701, 185702
**Valsartan**	247101, 247102, 247103, 247104
**Fimasartan**	515201, 515202, 515203

**Azilsartan**	662401, 662402, 662403
**Telmisartan**	378801, 378802
**Eprosartan**	429201
**Olmesartan**	468501, 468502, 468503, 520901, 520902
**Valsartan + Amlodipine**	492800, 492900, 495800, 522600, 522700, 522800, 522900, 523000, 523100, 523200, 523300, 523400
**Valsartan + Lercanidipne**	522200. 522300. 522400
**Valsartan + Pitavastatin**	634900, 635000, 635100, 635200
**Valsartan + Sacubitril**	651401, 651402, 651403
**Valsartan + Rosuvastatin**	629700, 629800, 525000, 525100, 525200, 525300,
**Valsartan + Hydrochlorothiazide**	356400, 442600
**Olmesaetan + Amlodipine**	500500, 500600, 547500. 547600, 547700, 547800, 547900, 548000, 582200, 582400, 629400, 629500, 629600, 631300, 632800, 632900, 633000
**Olmesartan + Hydrochlorothiazide**	513600
**Olmesartan + Hydrochlorothiazide + Amlodipine**	519700, 519800, 519900, 520000, 520100
**Olmesartan + Rosuvastatin**	653200, 644100, 644200, 526300, 526400, 526500, 526900
**Telmisartan + Hydrochlorothiazide**	502600, 443200, 443300
**Telmisartan + Rosuvastatin**	629900, 630000, 630100, 630200, 631600, 631700
**Telmisartan + Amlodipine**	511500, 511600, 511700, 521200, 521300, 521400, 623100, 644800
**Telmisartan+ Hydrochlorothiazide + Amlodipine**	663500, 663600, 663700, 663800
**Telmisartan + Rosuvastatin + Amlodipine**	671700, 671600, 671500, 671400, 671300, 671200,
**Losartan + Hydrochlorothiazide**	262500, 378900, 486900
**Losartan + Amlodipine**	502700, 503000, 513900, 637400, 637500, 637600
**Losartan + Rosuvastatin+ Amlodipine**	663900, 664000, 664100, 664200, 664300, 664400,
**Losartan + Hydrochlorothiazide+ Amlodipine**	662800, 662900, 663000
**Fimasartan + Hydrochlorothiazide**	522000, 526800
**Fimasartan + Amlodipine**	651900, 652000, 652100, 652700, 651900
**Fimasartan + Rosuvastatin**	654600, 654700, 654800, 654900, 655000
**Candesartan + Hydrochlorothiazide**	423700

**Candesartan + Amlodipine**	652900, 653000, 653100, 652900, 652900
**Candesartan + Rosuvastatin**	673700, 661800, 661900, 662000, 662100
**Irbesartan + Hydrochlorothiazide**	385700, 385800
**Irbesartan + Atorvastatin**	527000, 527100, 524000, 524100
**Azilsartan + Chlorthalidone**	673500, 673600
**Eprosartan + Hydrochlorothiazide**	460500
**Amlodipine + Atorvastatin**	614500, 472300, 472400, 472500, 518900
**Amlodipine + Rosuvastatin**	673900, 674000, 674100
**Atorvastatin + Ezetimibe**	633800, 633900, 634800
**Pitavastatin + Fenofibrate**	679300
**Rosuvastatin + Ezetimibe**	640700, 640800, 640900
**Metformin + Atorvastatin**	671800, 673800, 671900, 672000, 672100
**Metformin + Rosuvastatin**	672500, 672600, 672700, 672800, 672900, 673000, 683300, 683400
**Gemigliptin + Rosuvastatin**	664600, 664700, 664800
**Aspirin**	110701, 110702, 110801, 110802, 111001, 111001, 111001, 111002, 111003, 111003
**Clopidogrel**	133201, 133201, 133201, 133202, 133203, 506100
**Cilostazol**	136901, 492501, 495201, 498801, 501501
**Ticlopidine**	498900, 239201, 239202
**Aspirin + Bethocarbamol**	256800
**Aspirin + Clopidogrel**	517900, 517900, 517900, 667500
**Aspirin + Dipyridamole**	489700
**Atorvastatin**	111502, 502202, 633900, 472400, 518900, 524100, 527000, 672000, 672100, 111503, 502203, 634800, 472500, 111504, 502204
**Fluvastatin**	162401, 162402, 162403
**Lovastatin**	185801
**Pitavastatin**	470901, 470902, 470903
**Pravastatin**	216601, 216602, 216603, 216604
**Rosuvastatin**	454001, 454002, 454002, 454003, 454003, 454005

**Simvastatin**	227801, 227802, 227803, 227805, 227806
**Darbepoetin α**	455738, 455739, 455735, 455736, 455737, 455702, 455703, 455704, 455707, 455708
**Erythropoietin**	500334, 500337, 500340, 500341, 500342, 500343, 500330, 500332, 500331, 500333, 500335, 500338, 500336, 500339, 154305, 154308, 154303, 154306, 154309, 154301, 154302, 500301, 500302, 500303, 500304, 500305, 500306, 500307, 500308
**Methoxyl polyethylene glycol-epoetin β**	504302, 504303, 504301, 504305, 504304, 504306, 504307, 504308, 504309, 504311, 504310
**Paricalcitol**	430703, 430702, 430701, 430730, 430731
**Alfacalcidol**	104601, 104601, 104602
**Calcitriol**	121601, 121602, 121630
**Calcifediol**	121401, 121402
**Cinacalcet**	512301, 512302
**Cholecalciferol + Calcium carbonate**	302600, 387900, 409100, 473800, 480200, 498200, 498300, 526100, 634000, 521900
**Cholecalciferol + Calcium citrate**	462700, 462800, 519000, 503500, 504400, 508700, 665600, 670000
**Cholecalciferol + Calcium citrate**	503100
**Ergocalciferol + Calcium gluconate+ Calcium phosphate**	473300
**Ergocalciferol + Calcium carbonate+ Calcium gluconate + Calcium lactate**	303200
**Cholecalciferol + Alendronate**	481100, 500200
**Cholecalciferol + Ibandronate**	523900
**Cholecalciferol + Risendronate**	511200, 518400
**Calcitriol + Alendronate**	468000
**Cholecalciferol + Ralxifene**	659200, 659200
**Cholecalciferol + Bazedoxifene**	674500

Table S2. Erythropoietin resistance index and HR of cardiovascular events according to subgroups

	Univariable		Multivariable			Univariable		Multivariable	
	HR (95% CI)	*P*	HR (95% CI)	*P*		HR (95% CI)	*P*	HR (95% CI)	*P*
**Males**					**Females**				
Reference: Q1									
Q2	1.02 (0.97–1.08)	0.471	1.04 (0.96–1.12)	0.381		1.00 (0.92–1.09)	0.956	1.04 (0.94–1.15)	0.457
Q3	1.00 (0.94–1.06)	0.916	1.03 (0.93–1.15)	0.573		0.99 (0.92–1.07)	0.859	1.06 (0.93–1.20)	0.375
Q4	1.06 (0.99–1.13)	0.066	1.07 (0.91–1.25)	0.445		1.06 (0.98–1.14)	0.134	1.15 (0.96–1.38)	0.134
Reference: Q2									
Q3	0.98 (0.92–1.04)	0.436	1.00 (0.92–1.07)	0.918		0.99 (0.92–1.07)	0.805	1.02 (0.93–1.12)	0.705
Q4	1.04 (0.97–1.11)	0.246	1.03 (0.91–1.16)	0.647		1.06 (0.98–1.14)	0.127	1.11 (0.96–1.27)	0.155
Reference: Q3									
Q4	1.06 (0.99–1.14)	0.066	1.03 (0.94–1.14)	0.503		1.07 (0.99–1.14)	0.061	1.09 (0.98–1.20)	0.119
**< 65 years**					**≥ 65 years**				
Reference: Q1									
Q2	0.97 (0.91–1.04)	0.387	0.98 (0.90–1.07)	0.676		1.01 (0.94–1.08)	0.800	1.05 (0.96–1.15)	0.244
Q3	0.96 (0.90–1.03)	0.269	1.01 (0.90–1.14)	0.823		0.94 (0.87–1.00)	0.056	1.01 (0.90–1.14)	0.847
Q4	0.98 (0.92–1.05)	0.628	1.02 (0.86–1.21)	0.816		0.99 (0.93–1.06)	0.874	1.12 (0.95–1.33)	0.184
Reference: Q2									
Q3	0.99 (0.93–1.06)	0.807	1.03 (0.95–1.12)	0.453		0.93 (0.87–0.99)	0.028	0.96 (0.88–1.04)	0.335
Q4	1.01 (0.95–1.08)	0.722	1.04 (0.91–1.18)	0.554		0.99 (0.92–1.05)	0.674	1.07 (0.94–1.21)	0.333
Reference: Q3									
Q4	1.02 (0.95–1.09)	0.554	1.01 (0.91–1.11)	0.885		1.06 (0.99–1.14)	0.073	1.11 (1.01–1.23)	0.038
**Low CCI (<7)**					**High CCI (≥7)**				
Reference: Q1									
Q2	0.95 (0.88–1.03)	0.197	0.94 (0.84–1.04)	0.223		1.04 (0.98–1.10)	0.186	1.09 (1.01–1.17)	0.031
Q3	0.93 (0.86–1.01)	0.073	0.96 (0.83–1.10)	0.528		1.02 (0.96–1.08)	0.608	1.09 (0.98–1.20)	0.107
Q4	1.00 (0.93–1.09)	0.908	0.98 (0.80–1.20)	0.810		1.03 (0.97–1.09)	0.318	1.18 (1.02–1.37)	0.031
Reference: Q2									
Q3	0.98 (0.91–1.06)	0.619	1.02 (0.93–1.13)	0.675		0.98 (0.92–1.04)	0.425	1.00 (0.93–1.07)	0.972
Q4	1.06 (0.98–1.15)	0.164	1.04 (0.89–1.21)	0.606		0.99 (0.94–1.05)	0.754	1.08 (0.97–1.21)	0.158
Reference: Q3									

Q4	1.08 (0.99–1.17)	0.059	1.02 (0.91–1.15)	0.751		1.01 (0.96–1.08)	0.631	1.09 (0.99–1.18)	0.062
**Low Hb (<10)**					**Low Hb (≥10)**				
Reference: Q1									
Q2	1.03 (0.86–1.24)	0.722	1.07 (0.87–1.32)	0.522		1.00 (0.95–1.05)	0.984	1.03 (0.96–1.10)	0.432
Q3	1.00 (0.85–1.18)	0.984	1.00 (0.80–1.25)	0.986		0.96 (0.92–1.01)	0.153	1.04 (0.95–1.13)	0.443
Q4	1.01 (0.87–1.17)	0.876	1.07 (0.81–1.42)	0.644		1.01 (0.96–1.07)	0.594	1.11 (0.97–1.27)	0.138
Reference: Q2									
Q3	0.97 (0.84–1.12)	0.682	0.94 (0.79–1.11)	0.448		0.96 (0.92–1.01)	0.149	1.01 (0.95–1.07)	0.756
Q4	0.98 (0.86–1.12)	0.761	1.00 (0.80–1.24)	0.986		1.01 (0.96–1.07)	0.608	1.08 (0.98–1.19)	0.140
Reference: Q3									
Q4	1.01 (0.91–1.13)	0.854	1.07 (0.91–1.25)	0.432		1.05 (0.99–1.11)	0.066	1.07 (0.99–1.16)	0.089
**Low ESA dose**					**High ESA dose**				
Reference: Q1									
Q2	0.99 (0.94–1.04)	0.755	1.01 (0.93–1.11)	0.753		1.61 (0.72–3.60)	0.245	1.21 (0.54–2.71)	0.646
Q3	0.93 (0.85–1.01)	0.086	1.03 (0.90–1.19)	0.639		1.52 (0.68–3.38)	0.307	1.20 (0.53–2.69)	0.664
Q4	0.83 (0.49–1.40)	0.477	0.63 (0.33–1.19)	0.153		1.58 (0.71–3.52)	0.262	1.32 (0.58–3.00)	0.505
Reference: Q2									
Q3	0.94 (0.86–1.02)	0.133	1.02 (0.92–1.13)	0.711		0.94 (0.87–1.02)	0.140	0.99 (0.90–1.09)	0.839
Q4	0.83 (0.49–1.41)	0.496	0.62 (0.33–1.16)	0.134		0.98 (0.91–1.06)	0.640	1.09 (0.96–1.24)	0.164
Reference: Q3									
Q4	0.89 (0.52–1.51)	0.668	0.61 (0.32–1.14)	0.119		1.04 (0.99–1.09)	0.108	1.10 (1.02–1.19)	0.013
**Short HDV (<40 M)**					**Long HDV (≥40 M)**				
Reference: Q1									
Q2	0.97 (0.91–1.04)	0.370	1.00 (0.93–1.07)	0.940		1.04 (0.97–1.12)	0.242	1.01 (0.94–1.10)	0.727
Q3	0.96 (0.90–1.02)	0.189	0.97 (0.86–1.08)	0.553		0.99 (0.93–1.06)	0.761	1.00 (0.89–1.12)	0.995
Q4	1.03 (0.96–1.10)	0.452	1.02 (0.89–1.16)	0.825		1.02 (0.96–1.09)	0.477	1.00 (0.87–1.15)	0.990
Reference: Q2									
Q3	0.99 (0.92–1.05)	0.656	0.97 (0.88–1.06)	0.508		0.95 (0.89–1.02)	0.139	0.99 (0.89–1.09)	0.783
Q4	1.06 (0.99–1.13)	0.107	1.02 (0.91–1.14)	0.750		0.98 (0.92–1.05)	0.619	0.99 (0.88–1.11)	0.822
Reference: Q3									
Q4	1.07 (1.00–1.15)	0.046	1.05 (0.97–1.13)	0.210		1.04 (0.97–1.11)	0.304	1.00 (0.93–1.08)	0.989

Multivariate analysis was adjusted for age; sex; body mass index; vascular access type; diabetes; hemodialysis vintage; CCI score; ultrafiltration volume; Kt/V_{urea}; hemoglobin, serum albumin, serum creatinine, serum phosphorus, and serum calcium levels; renin-angiotensin system blockers, statin, clopidogrel, or aspirin use; presence of atrial fibrillation or cerebrovascular accidents; ESA dose per week; erythropoietin resistance index; transferrin saturation rate; and ferritin levels.
Abbreviations: Q1, first quartile; Q2, second quartile; Q3, third quartile; Q4, fourth quartile; CCI, Charlson Comorbidity Index; CI, confidence interval; HR, hazard ratio; Kt/V_{urea}, dialysis adequacy; Low CCI (<7), subgroup with CCI score <7; High CCI (≥7), subgroup with CCI score ≥7; Low Hb (<10), subgroup with hemoglobin level <10 g/dL; High Hb (≥10), subgroup with hemoglobin level ≥10 g/dL; Low ESA dose, subgroup with mean erythropoiesis-stimulating agent dose <5,660 IU/week; High ESA dose, subgroup with mean erythropoiesis-stimulating agent dose ≥5,660 IU/week; Short HDV (<40M), subgroup with dialysis duration < 40 months; Long HDV (≥40M), subgroup with dialysis duration ≥ 40 months.

Table S3. Baseline characteristics using a balanced cohort

	**Q1 (n = 34,043)**	**Q2 (n = 34,346)**	**Q3 (n = 34,440)**	**Q4 (n = 36,142)**	***P*-value**
Age (years)	60.4 ± 0.2	60.4 ± 0.1	60.8 ± 0.1	60.9 ± 0.2	0.792
Sex (male, %)	20,126 (59.1%)	20,349 (59.2%)	19,542 (56.7%)	18,071 (50.0%)	<0.001
HD vintage (months)	60 ± 1	60 ± 1	59 ± 1	62 ± 1	0.382
BMI (kg/m^2)	22.4 ± 0.0	22.4 ± 0.0	22.3 ± 0.0	22.1 ± 0.0	0.693
Diabetes (%)	15,731 (46.2%)	15,463 (45.0%)	15,099 (43.8%)	17,331 (48.0%)	<0.001
CCI score	6.87 ± 0.04	6.80 ± 0.03	6.84 ± 0.03	6.90 ± 0.04	0.147
Arteriovenous fistula (%)	29,069 (85.4%)	29,462 (85.8%)	29,423 (85.4%)	31,007 (85.8%)	0.102
Kt/V_{urea}	1.52 ± 0.00	1.53 ± 0.00	1.53 ± 0.00	1.53 ± 0.00	0.748
UFV (L/session)	2.29 ± 0.01	2.27 ± 0.01	2.87 ± 0.01	2.33 ± 0.01	0.287
Hemoglobin (g/dL)	10.6 ± 0.0	10.6 ± 0.0	10.6 ± 0.0	10.6 ± 0.0	0.635
Serum albumin (g/dL)	3.99 ± 0.00	3.99 ± 0.00	3.98 ± 0.00	3.97 ± 0.00	0.855
Serum phosphorus (mg/dL)	4.93 ± 0.02	4.94 ± 0.01	4.95 ± 0.01	4.96 ± 0.02	0.646
Serum calcium (mg/dL)	8.92 ± 0.01	8.91 ± 0.01	8.91 ± 0.01	8.93 ± 0.01	0.801
Serum creatinine (mg/dL)	9.46 ± 0.04	9.54 ± 0.03	9.52 ± 0.03	9.53 ± 0.04	0.092
Use of RASB (%)	23,559 (69.2%)	23,866 (69.5%)	24,494 (71.1%)	23,689 (65.5%)	<0.001
Use of aspirin (%)	4,575 (13.4%)	4,387 (12.8%)	4,390 (12.7%)	4,244 (11.7%)	0.603
Use of clopidogrel (%)	2,712 (8.0%)	2,774 (8.1%)	2,698 (7.8%)	2,517 (7.0%)	0.852
Use of statins (%)	14,543 (42.7%)	14,527 (42.3%)	14,270 (41.4%)	12,950 (35.8%)	<0.001
MI or CHF (%)	13,307 (39.1%)	13,272 (38.6%)	13,660 (39.7%)	13,333 (36.9%)	0.088
Atrial fibrillation (%)	2,562 (7.5%)	2,443 (7.1%)	2,483 (7.2%)	2,564 (7.1%)	0.377
CVA (%)	9,089 (26.7%)	9,209 (26.8%)	8,894 (25.8%)	8,471 (23.4%)	0.396
Transferrin saturation (%)	35.0 ± 0.4	34.7 ± 0.3	34.3 ± 0.3	34.4 ± 0.4	0.583
Ferritin (ng/mL)	270 ± 4	265 ± 3	268 ± 3	271 ± 3	0.312
ESA dose (IU/week)	3.7 ± 0.0	7.5 ± 0.0	11.0 ± 0.0	17.2 ± 0.0	<0.001

Data are expressed as means ± standard error for continuous variables and numbers (percentages) for categorical variables. *P*-values were tested using a general linear model with a complex survey design, incorporating sample weights. **Abbreviations:** Q1, first quartile; Q2, second quartile; Q3, third quartile; Q4, fourth quartile; BMI, body mass index; CCI, Charlson Comorbidity Index; CHF, congestive heart failure; CVA, cerebrovascular accident; HD, hemodialysis; ESA, erythropoiesis-stimulating agent; Kt/V_{urea}, dialysis adequacy; MI, myocardial infarction; RASB, renin-angiotensin system blocker; UFV, ultrafiltration volume.

Table S4. Erythropoietin resistance index and HR using a balanced cohort

	Univariable		Multivariable	
	HR (95% CI)	*P*-value	HR (95% CI)	*P*-value
**All-cause mortality**				
Reference: Q1				
Q2	0.99 (0.97–1.01)	0.340	1.00 (0.97–1.02)	0.843
Q3	1.03 (1.01–1.05)	0.007	1.05 (1.01–1.09)	0.005
Q4	1.10 (1.08–1.12)	<0.001	1.15 (1.10–1.19)	<0.001
Reference: Q2				
Q3	1.04 (1.02–1.06)	<0.001	1.05 (1.02–1.08)	<0.001
Q4	1.11 (1.09–1.13)	<0.001	1.15 (1.11–1.19)	<0.001
Reference: Q3				
Q4	1.07 (1.05–1.09)	<0.001	1.09 (1.07–1.12)	<0.001
**CVE**				
Reference: Q1				
Q2	0.99 (0.97–1.02)	0.504	1.00 (0.97–1.02)	0.773
Q3	0.97 (0.94–0.99)	0.006	0.97 (0.93–1.01)	0.090
Q4	0.97 (0.95–0.99)	0.013	0.98 (0.94–1.03)	0.384
Reference: Q2				
Q3	0.98 (0.95–0.99)	0.037	0.97 (0.94–1.00)	0.084
Q4	1.01 (0.98–1.03)	0.066	0.98 (0.95–1.02)	0.425
Reference: Q3				
Q4	1.00 (0.98–1.03)	0.832	1.01 (0.99–1.04)	0.288

Multivariate analysis was adjusted for age; sex; body mass index; vascular access type; diabetes; hemodialysis vintage; Charlson Comorbidity Index score; ultrafiltration volume; Kt/V_{urea}; hemoglobin, serum albumin, serum creatinine, serum phosphorus, and serum calcium levels; renin-angiotensin system blockers, statin, clopidogrel, or aspirin use; presence of myocardial infarction or congestive heart failure, atrial fibrillation, or cerebrovascular accidents; erythropoiesis-stimulating agent dose per week; transferrin saturation rate; and ferritin levels. **Abbreviations**: Q1, first quartile; Q2, second quartile; Q3, third quartile; Q4, fourth quartile; CI, confidence interval; CVE, cardiovascular event; HR, hazard ratio; Kt/V_{urea}, dialysis adequacy.

Table S5. Factors associated with an increase in ERI quartiles

	Coefficient	OR (95% CI)	*P*-value
Age (increase of 1 year)	0.012	1.01 (1.01-1.01)	<0.001
Sex (ref: male sex)	0.812	2.25 (2.14-2.38)	<0.001
HD vintage (increase of 1 month)	0.000	1.00 (1.00-1.00)	0.061
BMI (increase of 1 kg/m^2)	–0.162	0.85 (0.84-0.86)	<0.001
Diabetes (ref: non-diabetes)	–0.064	0.94 (0.91-0.97)	0.003
CCI score (increased score of 1)	0.021	1.02 (1.01-1.03)	<0.001
Vascular access (ref: arteriovenous fistula)	0.060	1.06 (1.00-1.13)	0.048
Kt/V_{urea}	–0.204	0.82 (0.74-0.90)	<0.001
UFV (increase of 1 L/session)	0.146	1.16 (1.13-1.19)	<0.001
Hemoglobin (increase of 1 g/dL)	–1.178	0.31 (0.30-0.32)	<0.001
Serum albumin (increase of 1 g/dL)	–0.229	0.80 (0.75-0.85)	<0.001
Serum phosphorus (increase of 1 mg/dL)	0.016	1.02 (1.00-1.03)	0.080
Serum calcium (increase of 1 mg/dL)	0.002	1.00 (0.98-1.03)	0.860
Serum creatinine (increase of 1 mg/dL)	0.038	1.04 (1.03-1.05)	<0.001
Use of RASB	0.450	1.57 (1.50-1.65)	<0.001
Use of aspirin	–0.062	0.94 (0.88-1.01)	0.082
Use of clopidogrel	0.006	1.01 (0.92-1.10)	0.893
Use of statins	–0.112	0.89 (0.85-0.94)	<0.001
MI or CHF	0.059	1.06 (1.01-1.11)	0.018
Atrial fibrillation	0.041	1.04 (0.96-1.13)	0.332
CVA	–0.089	0.92 (0.87-0.96)	0.001
Transferrin saturation (increase of 1 %)	–0.002	1.00 (1.00-1.00)	<0.001
Ferritin (increase of 1 ng/mL)	0.000	1.00 (1.00-1.00)	0.668

Results are expressed after multivariable ordinal logistic regression analysis, and all variables are included in the multivariable model. **Abbreviations:** BMI, body mass index; CCI, Charlson Comorbidity Index; CHF, congestive heart failure; CI, confidence interval; CVA, cerebrovascular accident; HD, hemodialysis; ERI, erythropoietin resistance index; Kt/V_{urea}, dialysis adequacy; MI, myocardial infarction; OR, odds ratio; RASB, renin-angiotensin system blocker; UFV, ultrafiltration volume.

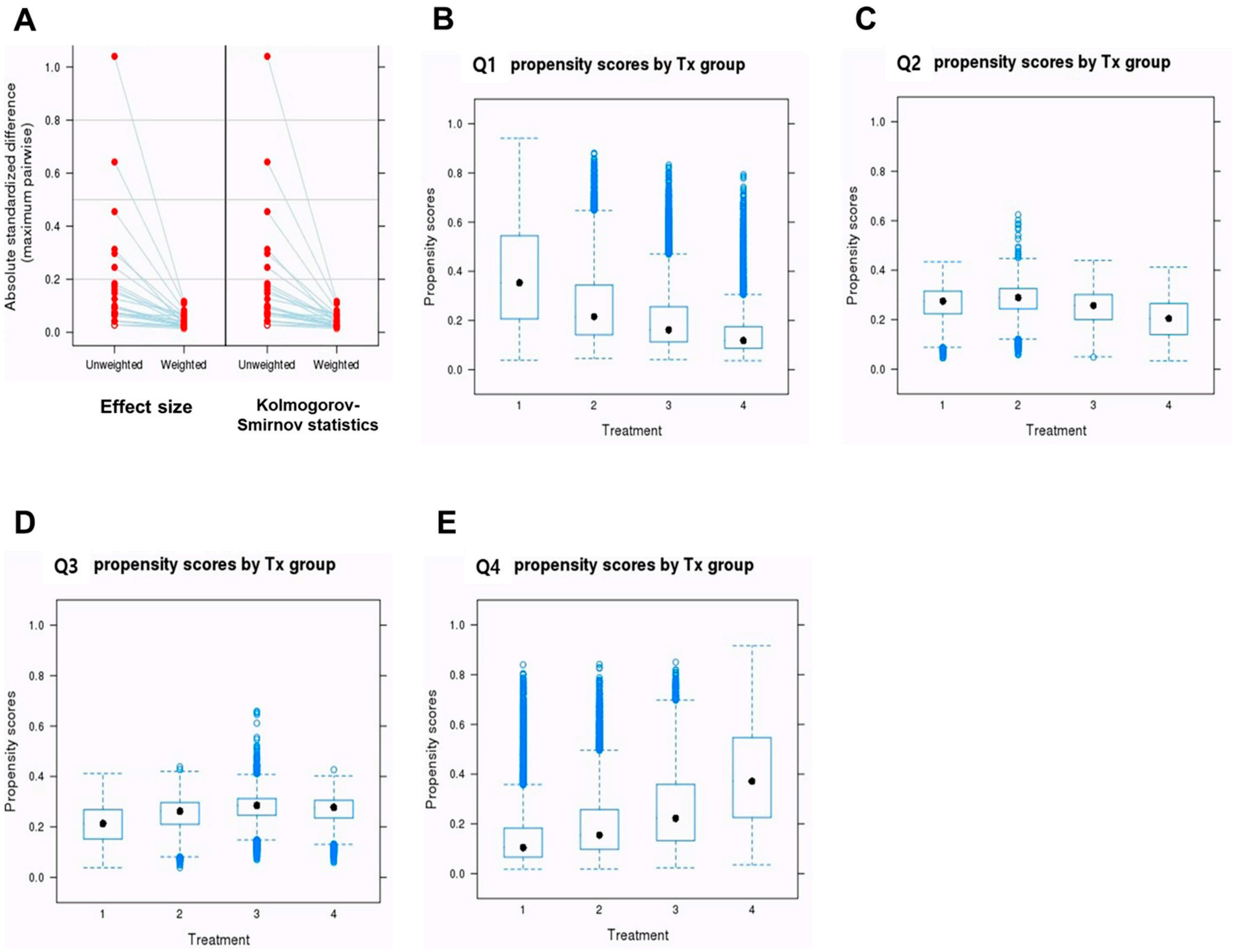

Figure S1. Balance tests. (A) Absolute standardized difference plots for estimating the propensity score to generate weights. (B–E) A boxplot illustrating the spread of propensity scores by treatment group for each group (i.e. Q1, Q2, Q3, or Q4 groups). The filled black circles indicate the median propensity score in each treatment group. As the plot demonstrates, there was substantial overlap in the total spread of propensity scores.

Abbreviations: Q1, first quartile; Q2, second quartile; Q3, third quartile; Q4, fourth quartile; Tx, treatment.

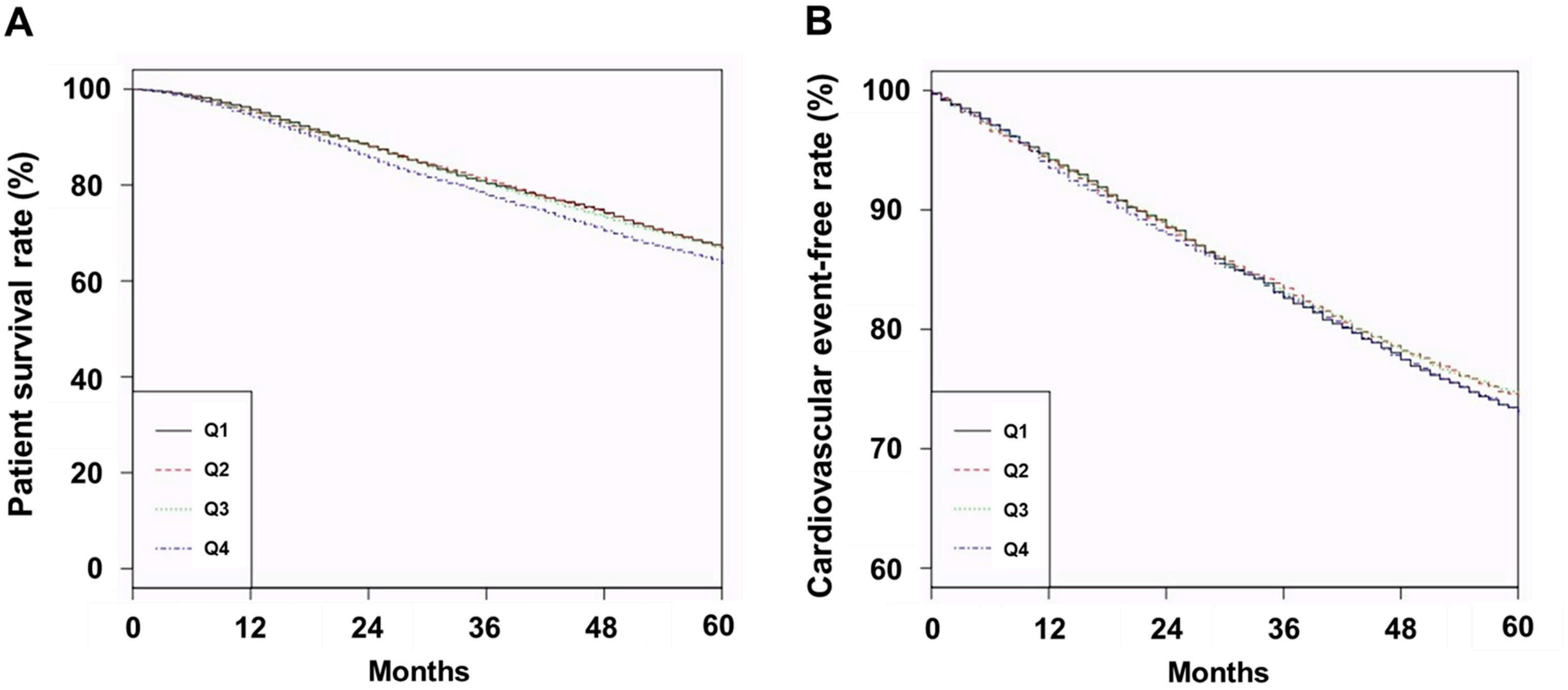

	Q1		
Q2	0.674	Q2	
Q3	0.222	0.073	Q3
Q4	<0.001	<0.001	0.005

	Q1		
Q2	0.420	Q2	
Q3	0.214	0.637	Q3
Q4	0.810	0.602	0.344

Figure S2. Kaplan-Meier curves by quartiles of the erythropoietin resistance index using a balanced cohort. (A) Patient survival and (B) cardiovascular event–free rates. *P*-values for pairwise comparisons with log-rank tests were added to the bottom of the graph.

Abbreviations: Q1, first quartile; Q2, second quartile; Q3, third quartile; Q4, fourth quartile.
